# Mechanism of the impacts of older adults social participation on their health

**DOI:** 10.3389/fpubh.2024.1377305

**Published:** 2024-08-07

**Authors:** Sheng Ai Lin, Xueqing Xu, Yutong Liu, Bin Ai

**Affiliations:** ^1^School of Management, Minzu University of China, Beijing, China; ^2^School of Ethnology and Sociology, Minzu University of China, Beijing, China

**Keywords:** older adults, social participation, health status, cohesion, influence mechanism

## Abstract

**Purpose:**

Against the background of population aging challenges in China, focusing on health, security, and social participation as core elements of positive aging, this study aims to formulate strategies for promoting the health of the older adults and reveal the pathways and trends of social participation in promoting health.

**Method:**

The study analyzed 1,015 randomly selected older adults individuals living at home in Beijing using household survey questionnaires. Drawing on group dynamics theory and structural equation modeling, the study proposed hypotheses regarding the relationships between social participation, group cohesion, and health status.

**Results:**

First, the triangular path model of social participation, group cohesion, and health status among the older adults was established. The direct path coefficient of social participation on health status was 0.15, that of social participation on group cohesion was 0.56, and that of group cohesion on health status was 0.32. The indirect path coefficient of social participation on health status through group cohesion was calculated at 0.56 × 0.32 = 0.18. Second, of the older adults age groups—younger, middle, and older—social participation’s direct path effects on health status were present only in the older age group. Social participation’s indirect path effects on health status through group cohesion were relatively high in all three groups, with a slight increase in the older age group.

**Conclusion:**

First, just the older adults participation in social activities serves as a benign stimulus to physical and mental health. Additionally, group cohesion formed through interaction with others during social activities encourages self-improvement behaviors, indirectly promoting health. In fact, indirect pathways of health promotion through group cohesion are stronger than direct pathways, highlighting the importance of group cohesion during social participation. Second, participation in activities alone can provide only sufficient benign stimuli for the older adults aged 80 and above, with the direct path effect of social participation on health beginning to appear only with increasing age. With age, selectivity of interaction with others decreases, and dependence increases; social participation’s indirect path effect on health through group cohesion continues to grow slightly.

## 1 Introduction

### 1.1 Research background and research questions

China’s population development has shown three major characteristics. In 2021, the proportion of people aged 65 and above in China exceeded 14% (1). In 2022, the number of births in China fell below 10 million for the first time (2). In 2022, China’s natural population growth rate was −0.60% (2). Therefore, in the context of intensified population aging and declining birth rates, as well as the overall population reaching its peak, the country, based on the concept of active aging, further emphasizes the social participation of the older adults in maintaining health and ensuring basic security. This includes organizing activities such as adult education, culture, sports, volunteering, and community governance (3). So, what is the promoting effect of social participation on health among the older adult? What is the promoting pathway of social participation on health among the older adult? What are the development trends of the promoting pathway of social participation on health among the older adult? This study aims to explore these questions through theoretical exploration and empirical research.

Social participation is an important way for older persons to integrate into society and participate in economic and social life, and its concept and connotation need to be further clarified. First, considering the group attributes of social participation, under activity theory, social participation is a group activity in which individuals participate (4); under resource theory, social participation is the sharing of individual resources into a collective (5); and interaction theory considers social participation to be the interaction of individuals in a group and with other members (6, 7). Therefore, it can be said that social participation is a group relationship that individuals establish with others in the process of participating in activities.

Second, regarding the spatial attributes of social participation, there is controversy over whether the social participation of the older adults includes household chores within the family (8) or is limited to social interactions outside the family (9). Social relations theory considers the social participation of older persons to be a source of social relations (10), and the social integration theory considers the social participation of older persons to be an important way for older persons to contact and relate to society (11). Therefore, the space for social participation should be external to the family, within the wider societal space.

Third, social participation has certain value attributes. According to the theory of social need, social participation of the older adults is the voluntary participation of individuals in activities that contribute to society (12); according to the theory of economic value, their participation refers to participation in productive activities (13), recent researchers conceptualized the social participation of the older adults as their ability to realize their own value and develop a higher sense of achievement (14). Therefore, social participation among the older adults encompasses not only social contributions and economic benefits but, more importantly, the attainment of a sense of personal worth for the older adults themselves.

To summarize, our research is based on group dynamics theory, utilizing survey data on the older adults population in Beijing and structural equation modeling. It defines older adults social participation as activities conducted outside the family, such as employment activities, volunteer work, recreational and sports activities within clubs or organizations, and interpersonal activities like communication with relatives and neighbors. The study will construct a path model to elucidate the relationship between older adults social participation, the group cohesion formed during social participation, and health promotion. The purpose of this study is to reveal the mechanism through which older adults social participation promotes health and the developmental trends of this mechanism with increasing age. This research intends to provide a foundation for developing more rational policies to promote older adults individuals’ health through social participation across varied age groups.

### 1.2 Literature review on pathways of promoting health through social participation among the older adults

#### 1.2.1 Research on the causal relationship between social participation and health among the older adults

There is a controversy regarding the causal relationship between social participation and health among the older adults. Numerous studies have revealed the positive effects of social participation on mortality risk (15), depression levels (16), and self-rated health (17) among the older adults. However, there are also studies indicating that health status significantly influences social participation (18). Therefore, some scholars have proposed that there is a mutual influence relationship between social participation and health among the older adults (19, 20). The latest research suggests that the impact of health status on social participation among the older adults is actually restrictive (21) and conditional (22), with health status merely providing possibilities for different levels of social participation (23).

There are three major theories on the strength of the impact of social participation on the health of older adults: health promotion theory, health damage theory, and the no-effect hypothesis. First, health promotion theory holds that social participation can enhance the health of older people, including improving subjective self-assessed health, increasing the sense of well-being, preventing geriatric diseases and dysfunctions, and reducing depressive moods (24–27). Second, the health damage hypothesis suggests that social participation can have negative effects on the health of older adults, including triggering depression and reducing life satisfaction (28, 29). Third, the no-effect hypothesis suggests that social participation has no significant effect on the health of older adults (30). That is, the academic community has not reached a consensus regarding the promotion effect of social participation on the health of the older adults.

#### 1.2.2 Research on the pathways through which social participation promotes health among the older adults

There are four main theories regarding the pathways through which social participation promotes health among the older adults. First, social participation reduces morbidity and injury by forming negative perceptions of aging, thereby lowering expectations. Role theory suggests that older people should gradually accept what is referred to as “compensatory roles” (31), thereby improving their sense of fulfillment (32). Second, social participation maintains health by perpetuating middle-aged lifestyles through the formation of positive perceptions of aging. Activity theory suggests that older adults should maintain the same pace of social participation as they did in midlife (33) and that more social participation can improve mental health and life satisfaction (34) or enhance well-being (35). Third, social participation promotes health by altering behavior through mutual restraint. Group dynamics theory suggests that the group dynamics formed through social participation drive older adults to develop healthier behaviors (36), participate in more physical activity (37), and develop healthier lifestyles (38). Fourth, social participation influences health through the dissemination of information. The group participation perspective suggests that high-quality health information dissemination improves public health information literacy and leads to healthier decision-making (39), whereas low-quality health information dissemination negatively affects the public’s health (40).

#### 1.2.3 The developmental changes of the pathways through which social participation promotes health among the older adults across the lifespan

Age discrimination is deeply embedded in institutions and cultures, for instance, the labor market’s attitudes toward older adults of various ages, which transitions from gradual acceptance to gradual exclusion and, eventually, complete exclusion (41). With increasing age, the selectivity of social participation patterns among the older adults decreases (42), implying that different age groups of older adults individuals participate in different social groups, each with distinct behavioral norms and values (43). Moreover, the ability of older adults individuals to participate in society varies significantly across different age groups (44), leading to differences in emotional experiences during the process of social participation among the older adults (45). Therefore, there are significant differences in the patterns, intensity, and emotional experiences of social participation among older adults of different age groups, suggesting that age stages may play a regulatory role in the pathways through which social participation promotes health.

#### 1.2.4 Issues not yet resolved in existing research

Existing studies have provided many empirical cases and theoretical perspectives on the intensity and mechanism of the impact of social participation on the health of older persons. However, with the continuous aging of the Chinese population and the development and changes in policies, culture, and spatiotemporal factors, there are still new urgent issues to be addressed regarding the path of promoting the health of the older adults through social participation.

First, the theoretical perspectives of existing studies have issues. Disengagement theory and role theory take a negative view of aging, arguing that older adults’ lack of or infrequent social participation contributes to their ill health. Activity theories view aging in a positive manner, believing that older adults should actively participate in society without discrimination. The above traditional gerontological theories all ignore the individual experience of the older adults in social participation. Meanwhile, the application research of group dynamics theory lacks research on pathway levels and direct and indirect pathway relationships.

Second, established empirical studies often lack relevant theoretical basis in the mediating variables the authors select, despite their statistical significance, the comparability with other similar studies is low, and the reliability of the research results is controversial.

Third, the literature lacks studies on developmental trends of pathways through which social participation promotes health across different stages of older adults’ lifespans. Indeed, the research has neither revealed the regulatory role of age stages in pathways through which social participation promotes health nor the developmental trends of these pathways’ mechanisms as age increases.

### 1.3 Theoretical perspectives and theoretical assumptions

Through field theory, Lewin found that changes in individual behavior are influenced by others and are the result of the joint action of intrinsic needs and extrinsic environment, proving the existence of group dynamics. Some researchers suggest that group dynamics can lead to interpersonal security (46) and increase an individual’s health behaviors (36). Therefore, this paper will build upon the theory of group dynamics to construct a theoretical pathway through which group dynamics formed during the process of social participation among older adults promote health and further develop research hypotheses.

#### 1.3.1 Theoretical construction

First, we explore the theoretical connotations and dimensions of group dynamics. Group dynamics refers to the social relations that form among groups or collectives (47) independent of the individuals, although they can have fundamental, direct, and extensive impacts on individuals (48). We expand the construct of group dynamics to five dimensions: cohesion, driving force, constraint, sense of belonging, and sense of security. Specifically, group cohesion reflects the centripetal force or solidarity among group members, driving the collective pursuit of group objectives (49). Driving force is the force or motivation that prompts group members to participate, act, and cooperate (50). Constraint establishes specific behavioral norms and expectations within the group (51). Belonging is the relationship of group members to other group members and their identification with the group (52). Sense of security is group members’ perception of their identity and status in the group, as well as their feelings about the stability and security of the group (53). Still, the relationship among these five dimensions of group dynamics is neither equal nor parallel. Cohesion, as the core of group dynamics, dictates the group’s cooperativeness and consistency. The driving force determines the group’s forward objectives, while constraint lays the foundation for cohesion. The sense of belonging and security are outcomes of cohesion. Given that this study does not focus on the organizational aspects of groups but rather on the path through which group dynamics, formed by the social participation of the older adults, promote health, it centers on group cohesion as the pivotal element of group dynamics, This focus aims to provide fresh theoretical insights into the relationship between social participation and health among the older adults.

Second, social participation plays a role in forming group dynamics (cohesion). Social participation has a group nature, connections individuals establish with each other as they participate in activities together (4), and cohesion is the result of group formation (54), so social participation has the condition of forming cohesion. Social participation is interactive. The spirit of mutual aid and cooperation and norms of mutual benefit can be formed in more interactive interactions (55), and cohesion is the force that keeps the group members coordinated and resistant to divisions (56). Therefore, there is consistency between the role of social participation and the formation of group cohesion.

Third, group cohesion plays a role in improving health conditions. On the one hand, group cohesion creates a sense of acceptance among group members (57) that enables older adults to develop new identities (58), enhance their sense of self, and create a sense of self-worth, which is beneficial to their physical and mental health. On the other hand, group cohesion can promote individuals’ responses to group members and the collective (59), thereby improving individuals’ physical and psychological states by influencing behavioral motivation, emotional communication, lifestyle, and other factors. Therefore, cohesion has a theoretical basis for acting on health.

#### 1.3.2 Research assumption

**Figure 1:**
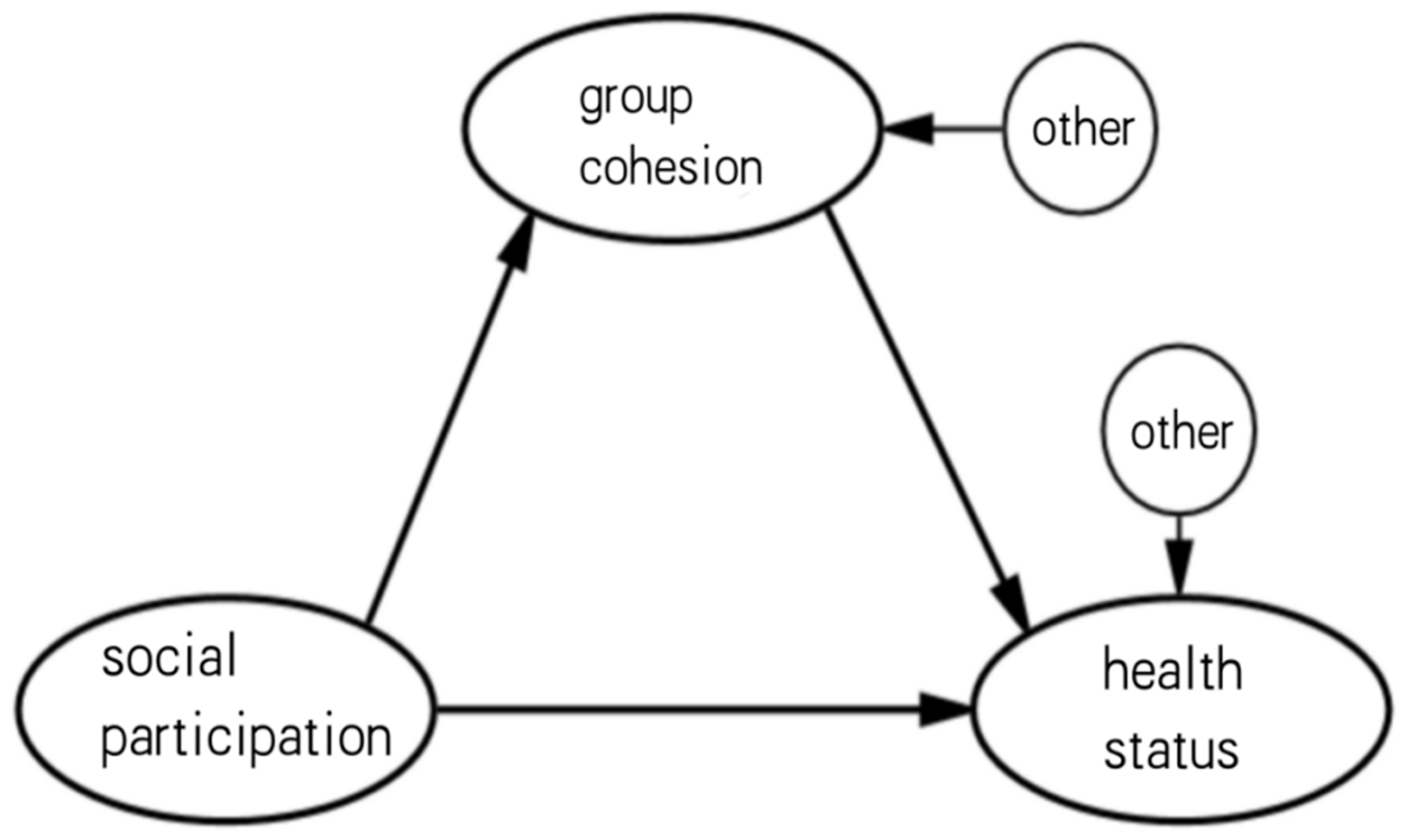
Hypothesized path relationships among social participation, group cohesion, and health status in older adults.

Based on the above theoretical synthesis, this study has constructed a triangular pathway model of the relationship between social participation, group dynamics (cohesion), and health (Figure 1) and proposes the following four research hypotheses.

*Hypothesis 1*: Older people’s social participation has a direct positive effect on their health as engaging in social activities in itself serves as a beneficial stimulus for physical and psychological well-being.

*Hypothesis 2*: Older people’s social participation leads to the formation of group cohesion, which in turn directly and positively impacts health. Through interactions with others in social activities, older adults continuously form various sizes and types of group cohesion, influencing individual behaviors.

*Hypothesis 3*: The effect of older people’s social participation on their health significantly varies by age group; the older the adults, the greater the positive effect of their social participation on their health mediated by cohesion.

*Hypothesis 4*: Direct and indirect pathways through which social participation promotes the older adults health vary with the life course. As older adults individuals age, their selectivity in social interactions decreases, leading to increased reliance on social participation to form group cohesion. For the older adults with declining physical strength, particularly, the mere act of participating in activities can provide sufficient positive stimuli for physical and mental well-being, thereby significantly increasing the direct pathway through which social participation promotes health.

## 2 Research methods

### 2.1 Research ideas

To elucidate the pathway mechanism through which social participation promotes health among the older adults, we first defined the research subjects as home-bound older adults aged 60 years and older, specifically urban older adults residents of Beijing, with the individual older adults as the unit of analysis. Second, we operationalized social participation as flexible employment, volunteering, membership in cultural and sports associations, and participation in neighborhood exchanges. Third, we conducted a survey to collect data from the participants on their social participation, group dynamics (cohesion), and health status. Subsequently, we used structural equation modeling to validate both direct and indirect pathways between social participation, cohesion, and health status among the older adults. Furthermore, we employed a multi-group analysis approach based on age strata to reveal the developmental trends of these pathway relationships.

### 2.2 Data collection methods

This survey was implemented by the Minzu University of China’s Research Group on the Mechanism of the Impact of Social Participation of the Elderly on their Health in cooperation with the Yongding Subdistrict Office of Haidian District. Considering the diversity in the composition and living environments of residents, the study selected two communities as typical survey sites: the community at No. 40 Fuxing Road on Yongding Road Street, predominantly consisting of retired older adults who were technologically skilled workers or government officials, and the Liujie Fang community, primarily composed of retired older adults workers and small vendors. From June to July 2023, a sample survey was conducted on 4,140 home-bound older adults individuals aged 60 and above residing in these two communities. To account for the differences between buildings within the same community and the similarities within each building, the study employed a cluster sampling method based on residential buildings. In the No. 40 Fuxing Road community, which comprises 16 residential buildings, we randomly selected buildings No. 2, 5, 8, 11, and 14. Similarly, in the Liujie Fang community, which consists of 20 residential buildings, buildings No. 4, 7, 10, 13, 16, and 19 were chosen at random. The investigators, consisting of community workers and university students, conducted household inquiry surveys. A total of 1,015 questionnaires were collected, resulting in a recovery rate of 84.58%. The analysis was conducted on data from 986 respondents after excluding incomplete questionnaires.

### 2.3 Measurement methods for core concepts

#### 2.3.1 Social participation measurement

In this study, social participation among the older adults is defined as their involvement in various forms of group activities in the social environment where they can realize their own value. Based on the health trajectory and social role of the older adults, social participation is divided into four levels: employment activities, voluntary activities, community organization activities, and neighborhood communication. Employment refers to working for an income for at least an hour, whereas voluntary activities encompass such public welfare activities as volunteer services or charitable assistance. Community organization activities involve various recreational and sports activities, and by neighborhood communication, we mean visiting or communicating face-to-face with neighbors.

#### 2.3.2 Group dynamics (cohesion) measurement

Cohesion is the result of group formation (54), which is the recognition by individuals of their belonging to a specific social group, as well as the significance of the emotions and values that group members bring to them (50). Hai et al. (60) employed qualitative survey data to establish the dimensions of cohesion, achieving consensus rates of 78.42, 67.48, and 71.43%, respectively, across different experts. They further validated the theoretical framework through sample and expert questionnaire surveys. Each dimension’s closed questions scored above 4 points, with a consensus rate of 71.43% for open-ended questions. Ultimately, they identified six dimensions for measuring cohesion: centripetal force, organizational strength, collaborative strength, solidarity, identification, and sharing. Based on this research, the current study operationalizes the concept of cohesion as follows: Centripetal force is represented by the willingness to join a group and become a member of it; organizational strength refers to recognizing and being willing to actively follow a group leader; collaborative strength is demonstrated through the commitment to task completion and mutual assistance; solidarity refers to maintaining interpersonal harmony in the group; identification is reflected by the alignment with group objectives. Lastly, the willingness to witness the group’s progress and partake in the benefits of its success represents the dimension of sharing.

#### 2.3.3 Health status measurement

Due to the specificity of health assessment in the older adults, it is not appropriate to simply use disease status for description. In this study, we operationalized health on three dimensions: self-care ability, life skills, and subjective health perception. Self-care ability was assessed by the ability to walk and bathe, life skills by the ability to shop and take medication, and subjective health perception by the individual’s perceived health status (Tables 1, 2).

**Table 1:** Distribution of health status characteristics of analyzed subjects.

					*n* (%)		
Core concepts	Measurement dimensions	Measurement dimension	Value	Option	Younger older adults	Middle-aged older adults	Older older adults
					244(24.7)	349(35.4)	393(39.9)
Health status	Self-care ability	Walking ability	1	Cannot at all	3(1.2)	8(2.3)	41(10.4)
			2	Very difficult	3(1.2)	1(0.3)	11(2.8)
			3	Quite difficult	1(0.4)	5(1.4)	20(5.1)
			4	Slightly Difficult	5(95.1)	15(4.3)	41(10.4)
			5	Not difficult	232(95.1)	320(91.7)	280(71.2)
		Bathing ability	1	Cannot at all	3(1.2)	5(1.4)	22(5.6)
			2	Very difficult	0(0.0)	3(0.9)	21(5.3)
			3	Quite difficult	1(0.4)	4(1.1)	17(4.3)
			4	Slightly difficult	3(1.2)	14(4.0)	33(8.4)
			5	Not difficult	237(97.1)	323(92.6)	300(76.3)
	Life skills	Shopping ability	1	Cannot at all	3(1.2)	9(2.6)	58(14.8)
			2	Very difficult	1(0.4)	8(2.3)	26(6.6)
			3	Quite difficult	0(0.0)	8(2.3)	29(7.4)
			4	Slightly difficult	3(1.2)	25(7.2)	47(12.0)
			5	Not difficult	237(97.1)	299(85.7)	233(59.3)
		Medication ability	1	Cannot at all	3(1.2)	4(1.1)	30(7.6)
			2	Very difficult	1(0.4)	4(1.1)	16(4.1)
			3	Quite difficult	1(0.4)	5(1.4)	37(9.4)
			4	Slightly difficult	9(3.7)	28(8.0)	65(16.5)
			5	Not difficult	230(94.3)	308(88.3)	245(62.3)
	Subjective health perception	Subjective health perception	1	Very poor	4(1.6)	7(2.0)	26(6.6)
	2	Poor	7(2.9)	16(4.6)	63(16.0)
			3	Fair	38(15.6)	87(24.9)	119(30.3)
			4	Good	70(28.7)	131(37.5)	120(30.5)
			5	Very good	125(51.2)	108(30.9)	65(16.5)

**Table 2:** Distribution of social participation and group cohesion characteristics among study participants.

					*n* (%)		
Core concepts	Measurement dimensions	Measurement dimension	Value	Option	Younger older adults	Middle-aged older adults	Older older adults
					244(24.7)	349(35.4)	393(39.9)
Social participation	Employment activity	Flexible employment	1	Never	212(86.9)	329(94.3)	373(94.9)
			2	Once or twice a month	8(3.3)	4(1.1)	3(0.8)
			3	Once or twice a week	4(1.6)	2(0.6)	4(1.0)
			4	Three or four times a week	6(2.5)	4(1.1)	3(0.8)
			5	Five or six times a week	14(5.7)	10(2.9)	10(2.5)
	Volunteer activities	Volunteer service	1	Never	117(48.0)	178(51.0)	267(67.9)
			2	Once or twice a month	70(28.7)	75(21.5)	51(13.0)
			3	Once or twice a week	21(8.6)	40(11.5)	35(8.9)
			4	Three or four times a week	12(4.9)	27(7.7)	16(4.1)
			5	Five or six times a week	24(9.8)	29(8.3)	24(6.1)
	Community organization activities	Cultural and sports associations	1	Never	110(45.1)	156(44.7)	258(65.6)
			2	Once or twice a month	45(18.4)	55(15.8)	57(14.5)
			3	Once or twice a week	30(12.3)	57(16.3)	27(6.9)
			4	Three or four times a week	24(9.8)	34(9.7)	22(5.6)
			5	Five or six times a week	35(14.3)	47(13.5)	29(7.4)
	Communication activities	Neighborly chat	1	Never	21(8.6)	27(7.7)	100(25.4)
			2	Once or twice a month	34(13.9)	29(8.3)	59(15.0)
			3	Once or twice a week	37(15.2)	54(15.5)	60(15.3)
			4	Three or four times a week	30(12.3)	71(20.3)	56(14.2)
			5	Five or six times a week	122(50.0)	168(48.1)	408(41.4)
Group cohesion	Centripetal force	Willingness to join	1	Strongly disagree	45(18.4)	76(21.8)	161(41.0)
			2	Disagree	20(8.2)	44(12.6)	47(12.0)
			3	Neither agree nor disagree	53(21.7)	65(18.6)	66(16.8)
			4	Agree	46(18.9)	75(21.5)	58(14.8)
			5	Strongly agree	80(32.8)	89(25.5)	61(15.5)
	Organizational strength	Recognition by leaders	1	Strongly disagree	47(19.3)	52(14.9)	134(34.1)
			2	Disagree	14(5.7)	46(13.2)	36(9.2)
			3	Neither agree nor disagree	58(23.8)	80(22.9)	89(22.6)
			4	Agree	55(22.5)	77(22.1)	68(17.3)
			5	Strongly agree	70(28.7)	94(26.9)	66(16.8)
	Collaborative strength	Collaboration and cooperation	1	Strongly disagree	44(18.0)	51(14.6)	126(32.1)
			2	Disagree	18(7.4)	34(9.7)	38(9.7)
			3	Neither agree nor disagree	53(21.7)	90(25.8)	88(22.4)
			4	Agree	59(24.2)	88(25.2)	72(18.3)
			5	Strongly agree	70(28.7)	86(24.6)	69(17.6)
	Solidarity	Interpersonal relationships	1	Strongly disagree	39(16.0)	41(11.7)	112(28.5)
			2	Disagree	14(5.7)	26(7.4)	28(7.1)
			3	Neither agree nor disagree	46(18.9)	73(20.9)	90(22.9)
			4	Agree	60(24.6)	99(28.4)	86(21.9)
			5	Strongly agree	85(34.8)	110(31.5)	77(19.6)
	Identification	Goal identification	1	Strongly disagree	41(16.8)	48(13.8)	123(31.3)
			2	Disagree	13(5.3)	25(7.2)	33(8.4)
			3	Neither agree nor disagree	51(20.9)	76(21.8)	87(22.1)
			4	Agree	66(27.0)	112(32.1)	82(20.9)
			5	Strongly agree	73(29.9)	88(25.2)	68(17.3)
	Sharing	Benefit sharing	1	Strongly disagree	41(16.8)	47(13.5)	120(30.5)
			2	Disagree	12(4.9)	25(7.2)	29(7.4)
			3	Neither agree nor disagree	49(20.1)	87(24.9)	87(22.1)
			4	Agree	74(30.3)	102(29.2)	76(19.3)
			5	Strongly agree	68(27.9)	88(25.2)	81(20.6)

### 2.4 Statistical and analytical methods

This study uses structural equation modeling for multi-cluster analysis. The theoretical hypotheses are presented as upstream and downstream path relationships between latent variables: social participation, group dynamics (cohesion), and health. After conducting analyses on the entire older adults population, individuals aged 60–69, 70–79, and 80 years and above are further categorized into three groups for multi-group comparative analysis. The three latent factors are represented using ellipses, and observed variables are represented using rectangles. The letters *d* and *e* indicate other unknown influences on each variable. The values on the arrows in each direction are standardized path coefficients ranging from −1 to 1, reflecting the magnitude and direction of the upstream variable’s impact on the downstream variable. The statistical analysis software used in this paper is SPSS 19.0 and Amos 19.0.

## 3 Analysis results

### 3.1 Evaluation of the fit of the path model for social participation, group cohesion, and health status among the older adults

**Figure 2:**
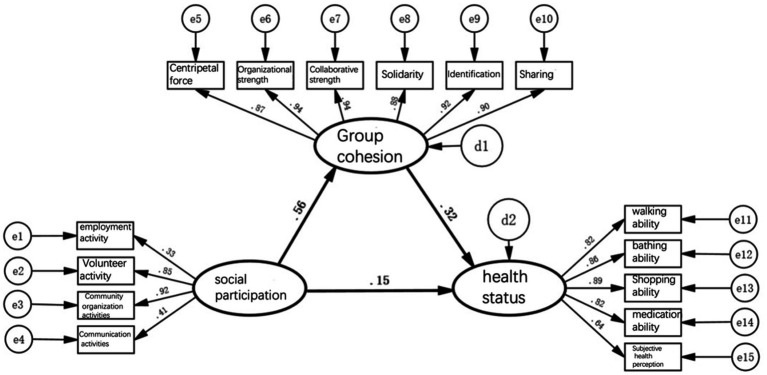
Model of the path relationship between social participation, group cohesion, and health status of all older adults.

**Figure 3:**
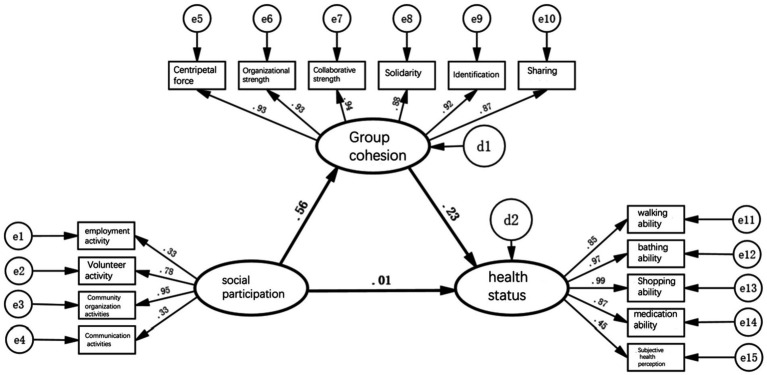
Pathway model of social participation, group cohesion, and health status of the younger older adults.

**Figure 4:**
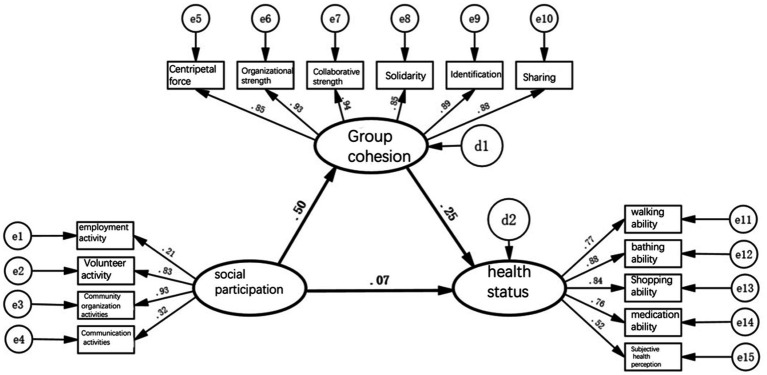
Pathway model of social participation, group cohesion, and health status of the middle-aged older adults.

**Figure 5:**
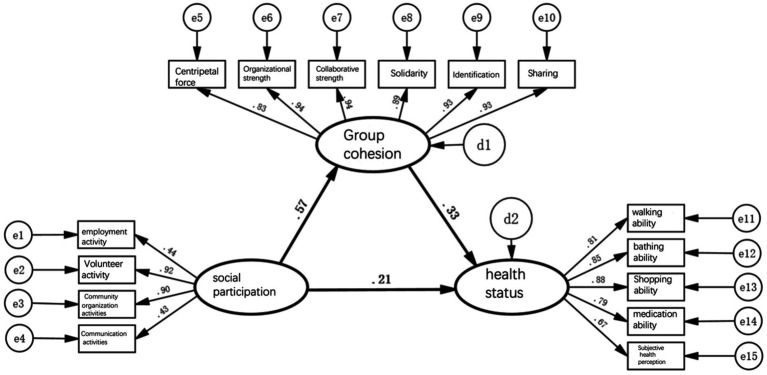
Pathway model of social participation, group cohesion, and health status of the older older adults.

Figures 2–5 depict path models illustrating relationships among social participation, group cohesion, and health status for the entire older adults population and also for the younger, middle-aged, and older groups, respectively. Model-fit evaluation criteria for structural equation modeling generally require PGFI greater than 0.5, NFI greater than 0.9, CFI greater than 0.9, and RMSEA less than 0.1. In this study, PGFI = 0.697, NFI = 0.922, CFI = 0.943, and RMSEA = 0.051, indicating that the path model has an ideal fit, falling within an ideal model’s range (61). Additionally, path models for the younger, middle-aged, and older older adults groups passed the test for factorial invariance, demonstrating the comparability of standardized path coefficients among the three groups (Table 3).

**Table 3:** Significance test of the non-standardized path coefficients for social participation, group cohesion, and health status among older adults.

variables	Younger older adults			Middle-aged older adults			Older older adults		
	Estimated value	Standard error	Probability	Estimated value	Standard error	Probability	Estimated value	Standard error	Probability
Social participation → Group cohesion	1.668	0.365	***	1.516	0.3	***	1.051	0.147	***
Social participation → Health status	0.016	0.086	0.855	0.086	0.087	0.327	0.322	0.103	**
Group cohesion → Health status	0.082	0.028	**	0.103	0.028	***	0.278	0.052	***
Social participation → Communication activities	1			1			1		
Social participation → Community organization activities	3.038	0.612	***	3.277	0.571	***	1.648	0.188	***
Social participation → Volunteer activity	2.173	0.435	***	2.588	0.445	***	1.583	0.181	***
Social participation → Employment activity	0.757	0.205	***	0.377	0.118	**	0.454	0.07	***
Group cohesion → Centripetal force	1			1			1		
Group cohesion → Organizational strength	0.981	0.035	***	1.021	0.041	***	1.114	0.043	***
Group cohesion → Collaborative strength	0.982	0.034	***	1.001	0.039	***	1.113	0.043	***
Group cohesion → Solidarity	0.915	0.039	***	0.883	0.043	***	1.043	0.045	***
Group cohesion → Identification	0.935	0.036	***	0.924	0.041	***	1.094	0.043	***
Group cohesion → Sharing	0.878	0.039	***	0.909	0.041	***	1.118	0.044	***
Health status → Walking ability	1			1			1		
Health status → Bathing ability	0.933	0.041	***	1.015	0.059	***	0.914	0.048	***
Health status → Shopping ability	0.994	0.042	***	1.334	0.081	***	1.245	0.061	***
Health status → Medication ability	0.934	0.052	***	0.894	0.062	***	0.918	0.052	***
Health status → subjective health perception	0.866	0.116	***	0.936	0.099	***	0.713	0.05	***

“*” indicates significance at the 0.05 level, “**” indicates significance at the 0.01 level, and “***” indicates significance at the 0.001 level.

### 3.2 Direct and indirect pathways of social engagement promoting health among older adults

First, in the path model for social engagement, group cohesion, and health status depicted in Figure 2, the path coefficient indicating the direct effect of social engagement on health status is 0.15; it thus passes the significance test, indicating that a change of 1 standard unit in social engagement can enhance health status by 0.15 standard units. This result confirms hypothesis 1, suggesting that participation of older adults in social activities is a benign stimulus process and directly improves their health status either physically or psychologically. However, the direct pathway’s health promotion is notably weak.

Second, in the model previously mentioned, the path coefficient indicating social engagement’s direct effect on group cohesion is 0.56, while that indicating group cohesion’s direct effect on health status is 0.32; thus, both pass significance tests. This implies that a change of 1 standard unit in social engagement can generate 0.56 standard units of group cohesion, and a change of 1 standard unit in group cohesion can result in 0.32 standard units of health status. This result validates hypothesis 2, indicating that older adults, by engaging in social activities, continuously form various attributes and sizes of group cohesion through collective interaction with others; this can also influence individual behavioral patterns.

Furthermore, in the above model, the path coefficient of social engagement indirectly affecting health status through group cohesion, calculated as the product of the two path coefficients, is 0.56 × 0.32 = 0.18, indicating that a change of 1 standard unit in social engagement can indirectly enhance health status by 0.18 standard units. This result verifies hypothesis 3, suggesting that the broader and more frequent social engagement of older adults, the stronger the group cohesion formed. This effect leverages external forces to improve behavior and lifestyle, consequently enhancing health status further. Compared with the direct pathway, the indirect pathway’s health promotion is relatively more significant.

Finally, social engagement’s comprehensive effect of promoting health among older adults, including direct and indirect pathways, amounts to 0.15 + 0.18 = 0.33, meaning that every change of 1 standard unit in social engagement can cumulatively enhance health by 0.33 standard units. These results underscore the significance of older adults forming strong group cohesion through social engagement as a crucial pathway for health promotion.

### 3.3 Changes in path through which social engagement promotes health among older adults with age progression

First, when the entire older population is divided into low, medium, and high-age groups for simultaneous multi-group analysis, the direct pathway effect of social engagement on health status is significant only in the high-age group model. Models for the low and medium age groups did not pass the significance test, indicating that the beneficial effect of older adults participating in social activities as a benign stimulus for improving health status applies only to those aged 80 and above. For the other age groups, merely engaging in activities is not sufficient to improve health status.

Second, the indirect pathway effects of social engagement on health status in models of the low, medium, and high age groups were 0.56 × 0.23 = 0.13, 0.50 × 0.25 = 0.13, and 0.57 × 0.33 = 0.19, respectively. They showed no significant difference, although in the high age group, the indirect effect increased slightly.

These results validate hypothesis 4, indicating that as older adults age, their selective reduction in interaction with others increases dependency. Consequently, the indirect pathway effect of social engagement promoting health through group cohesion increases. Additionally, as older adults age and experience declining physical strength, mere participation in activities can provide sufficient benign stimuli for their physical and mental well-being. Thus, the direct pathway effect of social engagement promoting health increases significantly.

## 4 Conclusion and discussion

### 4.1 Main conclusion

Against the backdrop of China’s proactive aging concept centered on health, security, and social participation to address the challenges of population aging, this study, based on social dynamics theory and utilizing structural equation modeling with survey data from older adults individuals in Beijing, reveals the pathway mechanism and developmental trends of how social engagement among older adults promotes health. The main conclusions are as follows:

First, social engagement, group cohesion, and health status form a triangular path structure, with social engagement influencing health status both directly and indirectly. Directly, older adults’ participation in activities itself improves health status slightly as a benign physical or psychological stimulus. Indirectly, social engagement fosters group cohesion, enabling older adults to leverage external forces to improve their health status.

Second, the direct path coefficient of social engagement on health status is 0.15, indicating a modest improvement in health status through older adults’ participation in social activities. Additionally, the direct path coefficient of social engagement on group cohesion is 0.56, suggesting that engaging in social activities fosters strong group cohesion through collective interaction.

Third, the indirect path coefficient of social engagement through group cohesion on health status is the product of the two path coefficients, 0.56 × 0.32 = 0.18. This indicates that higher levels of social engagement among older adults lead to stronger group cohesion, thus facilitating health status improvement by influencing behavioral and lifestyle changes. This impact of the indirect pathway on health is stronger than that of the direct pathway, highlighting the importance of group cohesion in health promotion among older adults during social engagement.

Fourth, while comparing age groups (low, medium, and high), the direct pathway effect of social engagement on health status is significant only in the high age group. However, the indirect pathway effect of social engagement on health status through group cohesion remains relatively high across all age groups, with a slight increase in the high-age group. Because participation in activities alone can sufficiently stimulate the physical and mental well-being of older adults aged 80 and above, the direct pathway effect of social engagement on health status significantly increases with age. Additionally, selective reduction in interaction with others among older adults increases dependency, indicating that the indirect pathway effect of social engagement on health status through group cohesion persists and increases slightly with age progression.

### 4.2 Discussion

#### 4.2.1 Mechanism discussion of the research findings

In the context of an aging population, a lack of understanding of the patterns of participation of older people has led to a lack of clarity about how social participation promotes health (61). When participating in social activities that are weakly regulated and lack goals, older adults people often tend to focus on their personal interests, neglecting the interests of the group and the needs of others and ultimately failing to bring about a positive impact on the participants. Therefore, to pay attention to the social participation of the older adults, not only should we pay attention to the frequency and intensity of the social participation of the older adults but also the mode of social participation of the older adults.

For older adults in good health, it is appropriate to participate in economic activities with formal norms and clear organizational goals. Second, for older adults in average health, it is appropriate to participate in volunteer activities that involve frequent interaction and solidarity with team members. Third, for older adults in slightly poorer health, it is appropriate to participate in community activities with periodic events and team members. Fourth, for older adults with worse health conditions, it is appropriate to participate in regular outings. The above social participation with a certain degree of periodicity, standardization, and purpose can have a positive impact on the health of the older adults.

When the older adults participate in organized group activities, the group cohesion formed can have an impact on them individually. First, when older people are able to live in harmony with other group members, a sense of collective efficacy is generated that further attracts older people to participate in group activities on a regular basis, gradually improving their behavior and forming a healthier lifestyle. Second, they can consciously pursue the group’s goals together with the group members, strengthen communication and cooperation with the team members in the process, and form close interpersonal relationships, which are conducive to the exchange and transmission of health information. Third, as the group identity is gradually formed, such group cohesion can directly affect individual behavior; people can be influenced by other members of the group to avoid unhealthy behaviors such as smoking and drinking.

There are three main reasons for the large differences in the pathways through which social participation promotes health between middle-aged and younger older adults versus older older adults. First, the older older adults have a greater likelihood of having a narrower social circle than the middle-aged and younger older adults. As they grow older, older people’s ability to move around is gradually restricted, making it more difficult for them to participate in social activities; moreover, as people age, they lose spouses, friends, and loved ones to illness, relocation, and death, making it easier for the older adults to develop social isolation and a sense of loneliness. Therefore, the oldest seniors obtain more emotional support from their social participation, and group cohesion forms more easily.

Compared with younger and middle-aged older adults, older older adults are more likely to have poor physical health. The risk of illness, cognitive decline, and depression is higher in the older adults of advanced age and is accompanied by declining immune system function as well as weakened physical functioning; periodic social participation makes it easier for mediating mechanisms to function in the context of relatively weaker physical and psychological functioning. It is possible that the increased positive health outcomes of social participation among the older adults result not only from the formation of group cohesion but also from behaviors that reduce social disengagement, such as active social interactions outside the home.

#### 4.2.2 Comparative analysis with existing studies

Firstly, previous studies propose that social participation reduces illness and injury by instilling a negative perception of aging. This perspective lowers expectations and gradually leads to acceptance of a “compensatory role” (31), thereby enhancing self-satisfaction (32). Our study, however, suggests that the group cohesion fostered through social participation enhances seniors’ positive outlook on life, leading to health improvements. This is especially true for older seniors, who also benefit from engaging in healthy social activities.

Secondly, earlier research suggests that maintaining the lifestyle from one’s middle age is crucial for preserving health, implying that seniors should consistently keep up with their social participation habits from their middle years (33). The current study counters this by arguing that forcibly maintaining the lifestyle from one’s middle age is not always feasible. Moreover, many individuals do not develop interests or hobbies during their middle years that are suitable for their senior years. Our research indicates that health benefits can be reaped simply by beginning to participate in community activities during one’s senior years.

Lastly, prior research posits that health is promoted by the restrictive changes in behavior patterns that stem from social participation. This suggests that the constraint of group dynamics pushes seniors towards adopting healthier behavior patterns (36), engaging in physical exercise (37), and maintaining healthy lifestyles (38). This study, however, underscores the importance of cohesion within group dynamics. These groups’ cooperative and consistent nature plays a more enduring and positive role in promoting health through social participation.

#### 4.2.3 Theoretical reflections on the research findings

First of all, the theoretical model applied in this study effectively explains the mechanism of the impact of social participation of the older adults on their health. Some scholars have explained the influence of group forces on the health of older adults based on the pathway of “community interaction environment → walking → health” (62). The authors of that study paid attention to the social interactions and group interactions of the older adults, but they only highlighted walking as the social interaction, which is unable to reflect the health behavior of the older adults in a comprehensive way. Moreover, it weakened the measurement effects of social interaction on older adults and failed to include the entire older adults population with regard to their health status in the study. In this paper, we use the theoretical perspective of group dynamics and propose the path of “social participation → group cohesion → health,” which can explain a variety of behavioral factors and include all levels of social participation of the older adults in the model. The model reflects the social activities that the older adults participate in more comprehensively, and at the same time, it can reflect the effect of social participation. In addition, older people at all levels of health can be included in the study, and older people in poorer health can also engage in certain social interactions, thus reflecting the effects of these social interactions on themselves.

Second, this paper introduces group dynamics theory to the topic of social participation of older people, incorporating the concept of group cohesion as a dimension of group dynamics in the model. In this study, six dimensions of cohesion were applied to measure cohesion, including individual, group, and organizational levels. Current cohesion scales are more often designed based on corporate, sports, or military contexts and less often based on the situation of older adults. In this paper, we refer to previous studies and rationalize the scale based on the situation of older adults in order to provide a tool for subsequent empirical research. In addition, group dynamics includes not only cohesion but also driving force, binding force, and other dimensions, and in the future, other dimensions of group dynamics can be measured and incorporated into this theoretical model to conduct further theoretical exploration and empirical testing of the explanatory mechanism of the impacts of older adults social participation on their health.

Finally, the findings of this paper demonstrate that older adults social participation can contribute to their health through the formation of group cohesion. In the past, older adults were often labeled as negative and weak; the retirement age policy excluded those aged 60 and above from the job market, and there was widespread discrimination against the older adults in the labor market. Policy arrangements, media discourse, and traditional notions of old age have led to a gradual disengagement of the older adults from the workplace. Many older adults themselves develop a sense of uselessness, which is detrimental to social development and their own health. Therefore, we should take positive aging as an important strategy for the management of aging in China. In this paper, we use group dynamics theory to explain the positive impact of social participation of the older adults on their health and analyze it from a positive perspective of aging. We should respect, encourage, and support the social participation of the older adults and pay attention to the mode of social participation of the older adults without neglecting the social value brought by the older adults.

National policies should support the social participation of the older adults, including improving the retirement age policy, strengthening job security for older adults workers, and building more places for older adults activities. The media should update their concepts and publicize the idea of positive aging to reduce the stereotypical image of the older adults in society as a whole. Communities should organize more activities for the older adults and provide more convenient activity spaces. This paper also analyzes the effect of social participation on the health of different older adults groups, revealing the pathway effect of group heterogeneity on the promotion of health through social participation among the older adults, and provides the basis for the formulation of more targeted social participation encouragement policies for different older adults groups.

## Data availability statement

The original contributions presented in the study are included in the article/supplementary material, further inquiries can be directed to the corresponding author.

## Ethics statement

Ethical approval was granted by the Biological and medical ethics committee, Minzu University of China (C2023015CO) for this study on human participants in accordance with local legislation and institutional requirements. Written informed consent from the participants OR participants legal guardian/next of kin was obtained prior to participation in this study in accordance with the national legislation and the institutional requirements.

## Author contributions

SL: Investigation, Writing – review & editing. BA: Supervision, Writing – review & editing. XX: Investigation, Writing – original draft. YL: Investigation, Writing – original draft.
